# Notes upon Dentine and Enamel

**Published:** 1896-07

**Authors:** Charles S. Tomes


ARTICLE II.

### NOTES UPON DENTINE AND ENAMEL.

BY CHARLES S. TOMES, M. A., F. R. S.

Read before the Odontological Society of Great Britain.

During the past year a very interesting series of papers have appeared in the *Dental Cosmos* from the pen of Dr Black, in which, amongst other things, he has endeavored to ascertain the nature of that difference between teeth of good and bad quality which is recognized as existent by every dentist of any experience.

The idea generally has prevailed that, just as occurs in rickety bones, there was a deficiency in lime salts in teeth of poor quality; and but little had been done in the way of actual experiment towards the determination of this point, although Dr. Black appears to have overlooked the valuable papers of Dr. Galippe, who was first in the field of experimenting.

Dr. Galippe's experiments were undertaken with whole teeth, which was in some degree unfortunate, as it was always possible that differences in some of the tissues might be more or less completely masked by their not being common to all; moreover, he adopted the method of ascertaining the specific gravities of the teeth, which, according to him, corresponded in a rough way with the proportions of lime salts present.

According to Dr. Galippe ("Recherches sur les Proprietes Physiques des Dents," Societe de Biologie, 1884), the milk teeth have a lower specific gravity than their successors, and also differ in containing a larger percentage of carbonates; the roots of teeth also have a less specific gravity than their crowns, and he also believes that their density increases with age. He also holds that teeth of the right side have a slightly greater density than those of the left, and molars a greater than incisors.

When Dr. Black's researches were undertaken, little beyond this was known as to the constitution of teeth, and they hence constitute a valuable contribution to the subject.

His investigations covered a great deal of other interesting ground, but for the present I am concerned with one aspect of the question only—namely, that of the chemical constitution of the teeth.

Dr. Black procured teeth from mouths where the teeth were conspicuously free from caries, and from others where the teeth were decidedly carious, and adopted a uniform method of examining them. He cut transverse slices from the necks of the teeth, considering that in that position his results would be but little interfered with by the presence of enamel or cementum, and that they would therefore give good comparative results.

The slices were dried for an adequate length of time at a temperature of 100 degrees C., and then incinerated, first on gauze and then in a platinum crucible and the loss of weight set down as organic matter.

I have elsewhere (*Journal of the British Dental Association*, 1895) given grounds for thinking that the technique adopted was not calculated to give the most accurate results which could have been obtained; nevertheless his results are set out in his tables with a degree of minuteness which far transcends the possibilities of experimental accuracy, even had the technique been as perfect as could be. Still the broad results are, I think, quite trustworthy, and, at all events, striking out the decimal places, may be accepted as accurate.

Dr. Black's results give as a general average 72 per cent of lime salts (leaving out as untrustworthy his second place of decimals), and, which at first strikes one as remarkable, he found no deficiency in lime salts in the teeth of imperfect quality. This led me to undertake a series of check experiments, endeavoring to eliminate every source of error of which I could think.

In order to get dentine, and dentine only, the teeth wers sawn across at their necks, and as much dentine as could be got without danger of contamination with enamel or cementum, cut out with spear-headed drills and collected as fine turnings.

These were dried in a constant temperature of over eight hours at 100 degrees C., weighed in a platinum crucible, ignited in a muffle, and weighed again. The ash was then moistened with ammonium carbonate, in order to restore any carbonic acid which might have been driven off by ignition from the carbonates present, then dried and weighed again. The general result was an average of 72.5 per cent of lime salts. In these experiments the turnings remained from first to last in the crucible, so that there was no chance of loss, and the amount of dentine obtainable from each tooth was quite twice as much as the quantity experimented with by Dr. Black, this, again, of course, tending to diminish error.

One of the most enterprising statements in Dr. Black's paper is that teeth taken from the same mouth differ in the percentage of lime salts more largely than do teeth of good and bad quality. This seemed so unlikely that I took four or five jaws in which all, or almost all, the teeth were present, and estimated the salts in each tooth, or in each pair of teeth. As I soon found that the two central incisors, two lateral, or any other pair or teeth gave pretty closely the same result, I am inclined to think that Dr. Black is mistaken in supposing that these differ, and that the difference is due to experimental error. I therefore in my later experiments mixed the turnings from each pair of teeth, so as to get a larger quantity to deal with. The following are some of my later results:

(1) A perfect dentition (upper jaws) in which M3 is not yet erupted. Percentage of lime salts.

| | |
|---|---|
| Central incisors . | 70.0. |
| Lateral incisors . . | 69.7. |
| Canines . , | 70.8. |
| Bicuspids . . | 72.0. |
| First molars . . | 72.7. |
| Second molars . . | 72.9. |

(2) A perfect dentition in which only one wisdom tooth is erupted.

| | |
|---|---|
| Central incisors (probable error) | 64.5. |
| Lateral incisors . | 72.9. |
| Canines . . | 72.8. |
| First bicuspids . | 74.8. |
| Second bicuspids . . | 74.8. |
| First molars : | 74.6. |
| Second molars . . | 74.5. |

(3) Perfect dentition, a good deal worn.

| | |
|---|---|
| Right central and lateral incisors | 70.5. |

| | |
|---|---|
| Left central and lateral . | 70.4. |
| Right canines . | 72.0. |
| Left canines . . | 72.0. |
| Right bicuspids . | 72.7. |
| Left bicuspids . . | 72.8. |
| Right molars . | 72.3. |
| Left molars . . | 72.3. |
| Right and left wisdom teeth | 71.8. |

(4) Nearly perfect lower dentition. Instead of making an ignition, the dentine turnings were subjected to the action of strong nitric acid, and heated so as to drive off the acid. This left a perfectly white ash, which was treated with ammonium carbonite, to restore any lost carbonic acid.

| | |
|---|---|
| Central and lateral incisors | 75.6. |
| Canines . . | 75.6. |
| Bicuspids . | 75.3. |
| First molars . . | 79.3. |
| Second molars . | 77.2. |
| Third molars . . | 77.0. |

It will be seen that these percentages are higher, probably owing to the retention of some water. As a matter of fact, ignition subsequently brought down the percentage of the molars to 73, but, owing to an accident to the muffle, the others were lost.

(5) The incisors much grooved; two bicuspids and one wisdom tooth deeply carious; slight interstitial caries upon many of the teeth.

| | |
|---|---|
| Central and lateral incisors | 70.9. |
| Canines . | 71.2. |
| Bicuspids . . | 71.2. |
| Molars (first) . | 72.1. |
| Second molar . . | 71.9, |
| Third molar . | 72.0. |

It will be noticed that in this, the only series of imperfect teeth examined, the percentages rule lower than in the others, and this, so far as it goes, does not confirm Dr. Black's conclusion that teeth of poor quality are quite as highly calcified as those of good. But, on the other hand, the differences are small, and a single series does not establish much, so that this question must be left still *sub judice*.

It is suggested by Dr. Black that the difference between good and bad teeth is, probably, to be sought in the organic matter; there are, however, other possibilities. Thus, it may be in the proportion of combined water, or in the proportion of the different lime salts present, as, for example, in a greater proportion of carbonates. This, however, is a point very difficult to determine, as the estimation of carbonates present in very small quantity is open to much experimental error. Or, again, it is conceivable that the dentine may be identical, but the enamels different.

But there is one point which my investigations bring out very plainly, and that is, that the molars and bicuspids are more highly calcified than the incisors and canines.

Taking the averages from all the ignitions which I have performed, there comes out this result:

| | Percentage of salts. |
|---|---|
| Incisors and canines . | 71.5. |
| Molars . . | 73.2. |

Upon returning to Dr. Black's published tables and getting out the incisors and molars, I find these averages:

| | |
|---|---|
| Incisors and canines . | 71.7. |
| Molars . . | 71.3. |

I should say that in making comparison with Dr. Black's figures, which are percentages of wet dentine, I have corrected them into percentages of dry dentine, as all chemists are in the habit of giving analyses of substances deprived of free water. Thus, though Dr. Black has not noticed it, his figures serve to confirm my result, and it may be taken as pretty well established, the more so as

Dr. Galippe's specific gravity experiments on entire teeth point to the same conclusion.

It would be interesting (and I intend to investigate the point) to know if this is general throughout the animal kingdom. But, as ivory is known to contain a specially low percentage of salts, I compared the salts contained in it and in the dentine of an elephant's molar, with the following result:

| | |
|---|---|
| Ivory (tusk) . . | 57.5. |
| Dentine of elephant molar | 70.2. |
| Cementum of elephant molar | 67.0. |

Thus, then, whilst ivory is very rich in organic matter and water, the dentine of the molar differs little from human dentine. The tusk, which requires elasticity, but is little subject to attrition, has few lime salts, and the dentine of the molar, which is required to be hard—harder than the cementum—is highly calcified; and the cementum stands between the two in this respect.

Passing from these examinations, which were all carried out upon the line of destruction of the organic matrix, I approached the matter from the opposite direction, and dissolved out all the salts by the careful use of dilute acids.

The decalcified matrix was carefully washed and dried and then weighed. This gave a result at first sight not consistent with the ignition experiments.

Thus in the case of Ivory:—

| | |
|---|---|
| Lime salts (ascertained by ignition) | 57.5. |
| Dry collagen . . | 34.0. |
| Deficiency . . | 8.5. |
| | 100.0. |

This deficiency was ascertained to be water in combination; that is to say, water which could not be dried out.

Other experiments gave as much as 9.5 per cent of water; if this latter figure be correct, there is an amount of water in ivory sufficient to form three equivalents in combination with the tri-basic calcium phosphate, the formula of which would be $Ca_3\ P_2\ O$, $3\ H_2O$.

It is of course well known that calcium phosphate prepared by wet processes always contains one or more equivalents of water, the last of which is only lost at red heat.

A similar course of procedure with human dentine gave—

| | |
|---|---|
| Salts . . . | 72.0. |
| Collagen . . | 19.6. |
| Deficiency (water) . . | 8.4. |
| | 100.0. |

Now one equivalent of water in combination with the calcium phosphate, supposing that all the 72 per cent of the salts were calcium phosphate, which of course they are not, would require 3.9 per cent, so that there is not enough to form three equivalents of water.

There are great discrepancies in analysis of dentine, but all agree that there is some carbonate, and some magnesium phosphate, reducing the percentage of calcium phosphate down to 67 or 68 per cent, or, according to some, even lower; if that be the true proportion of calcium phosphate, the water is not far off the quantity required for three equivalents.

Hence, I am inclined to believe that in dentine the calcium phosphate holds, in chemical combination, two or three equivalents of water. But the definite determination of this point would need more careful analyses of rather large quantities of dentine.

But at all events this is clear, that the analyses of dentine as usually set out are wrong, in that a good deal of water is set down as organic matter, and that instead of setting down the combination of ivory as—

| | |
|---|---|
| Lime salts, etc . . | 57,5. |
| Organic matter . . | 42.5 per cent. |

We ought to write—

| | | |
|---|---|---|
| Lime salts | . . | 57.5. |
| Organic matter | . | 34.0. |
| Water | . . . | 8.5. |

Or—

| | |
|---|---|
| Organic matter, and water in chemical combination. . . . | 42.5. |

The collagen obtained was boiled for some time in water acidulated with acetic or weak hydrochloric acid; this brought the collagen into solution, partly changing it into gelatin, and left an insoluble residue of flocculent appearance, which consisted, as determined by microscopic examination, of Neumann's sheaths in a detached condition.

This residue, consisting, as shown by chemical tests, of elastin, was found to constitute as much as 1.2 per cent of the entire dentine in the case of ivory, and therefore formed quite 3 per cent of the dried collagen.

Before leaving the subject of dentine, I may mention that some tooth-pulps from a calf were dried and ignited; the dried pulps were found to contain 10 per cent of lime salts, mostly phosphates; there is then in the pulp a rich store of lime to draw upon for the purpose of calcification.

Blood plasm contains about .8 per cent of salts, but these do not largely consist of phosphates; dried blood plasm would contain a total of about 8 per cent in round numbers.

My observations upon enamel have been already published, in the *Journal of Physiology*, but I may mention that I have found it to be practically an inorganic tissue; the small percentage which has usually been set down as organic matter being, in fact, mainly water, and the organic matter present being only in the most infinitesimal proportion—far too little for quantitative estimation.

Just as in the case of dentine, it is probable that the calcic phosphate has an equivalent of water combined with it, and this is driven off with such suddenness, just before red heat is reached, as to make the enamel fly about.

An average analysis of enamel from an elephant's molar would give—

| | |
|---|---|
| Lime salts, etc . . | 94.51. |
| Water and traces of organic matter | 5.49. |

Calcining enamel leaves its structure but little altered. If it be not carried beyond bright redness for ten minutes or thereabouts, the enamel prisms still remain quite distinct and unaltered in size or form. They do, however, acquire a greater opacity, and a slightly granular appearance, so that it seems as if the driving out of the combined water had altered the crystalline structure.

It has long been known that the shells of certain molluscs present a prismatic structure not unlike that of enamel, and the shell of the large mussel Pinna is a fine example of this.

Thinking that it might throw some side-light upon the structure of enamel, I examined the Pinna shell, in which the calcifying material is carbonate and not phosphate of lime, and in which the prisms are of great size.

The prisms of Pinna present a further resemblance to those of enamel in that the individual prisms show a transverse striation, which has been beautifully brought out in a photograph, which was taken by Mr. Mummery.

A careful examination of these large Pinna prisms seem to indicate that the striation is due to regularly recurring varicosities of the prism. This tends to confirm the view more recently advocated* as to the cause of striation in human enamel—a view which is adopted by Mr. Leon Williams, and is illustrated by the very beautiful micro-photographs which he has been kind enough to lend me for exhibition here this evening.

On decalcifying Pinna shell I found that the organic residue forms a conspicuous and bulky mass, swelling up considerably when the lime is removed.

Yet this conspicuous and bulky mass proves to amount to only 1 per cent of the weight of the shell, which is a

*"Dental Anatomy," Tomes, 1894, p. 17.

confirmation of the conclusion that there is practically no organic matter in enamel, which, as is well known, wholly disappears on decalcification. An analysis of Pina shell gives—

| | |
|---|---|
| Lime salts . . | 89.2. |
| Organic matrix . | 1.3. |
| Water . . | 9.5. |

The organic residue has the appearance under the microscope of a delicate connective tissue. In order to investigate more satisfactorily its arrangement, I ground a section transverse to the prisms, and cemented it down to a slide with Canada balsam.

This was carefully decalcified, a little ferric chloride being added to the fluid; after washing it was treated with tannin solution, with the result that a beautiful honeycomb, corresponding exactly to the appearance of a simply ground section, was obtained, except only that the strongly refractive material which constituted the prisms was wholly gone.

It was thus shown that the Pinna shell consists of crystalline prisms of calcic carbonate containing no organic matter in themselves, but deposited in a honeycomb of connective tissue.

I should have mentioned that in a transverse section of shell the interstitial substance between the prisms is of material width, and the interstitial substance between enamel prisms of course is not.

A section of enamel was then treated in precisely the same way, with the result that nothing whatever could be distinguished upon the slide—all had disappeared.

In the absence of definite knowledge, save that a trace of interstitial matter can be stained in sections of human enamel (Bodecker and Heitzmann), and that a similar tissue can be demonstrated in epithelium—moreover that a trace of mucin can be got from enamel, as I have shown in the paper referred to above—it seems a legitimate speculation that enamel may be formed on lines somewhat alalogous to the Pinna shell; namely, that the lime salts may be deposited in the interior of the enamel cells, and so a definite pattern produced, their exceedingly delicate walls playing the same part as the connective tissue honeycomb in the shell, and that the comparative absence of organic material in the finished product may be due to their exceeding tenuity as well as to the absence of organic matter from the prisms themselves, which are purely crystalline.

Such an explanation seems to me to fit in with all known facts, and serves to easily account for the complex arrangement of the prisms in definite patterns which would on ordinary excretion views, be a little difficult.

My own analyses, and this view if it be true, afford an explanation of enamel structure quite at variance with that held by Heitzmann and Bodecker (which indeed the analyses alone seem sufficient to refute), and they fit in well with the explanation offered by Klein of the microscopic appearances.

And although the subject requires much more investigation, it seems very possible that the difference between good and bad teeth may reside principally in the enamel. We know that enamel formation is very sensitive to health disturbances, inasmuch as these become written upon it in the form of lines of imperfect structure when acute illness has happened during its deposition, and we know also that in teeth of poor quality it is easily cut and is friable, so that we have a difficulty in getting good edges to our cavities. But while it is tempting to indulge in what the late Professor Tyndall called the scientific use of the imagination, I would not be understood to build too much upon anything which goes far beyond a demonstrable experimental basis

It is said by Dr. Galippe that entire teeth contain about .4 per cent of silica, but this point I have not so far investigated sufficiently to enable me to speak with any certainty. If, however, it be present, I am inclined to think that it is only in smaller traces.—*British Journal of Dental Science.*

